# Structure–activity relationships of the intramolecular disulphide bonds in LEAP2, an antimicrobial peptide from *Acrossocheilus fasciatus*

**DOI:** 10.1186/s12917-024-04106-8

**Published:** 2024-06-04

**Authors:** Ci-Gang Yu, Li Ma, Di-Ni Zhang, Yue Ma, Chang-Yong Wang, Jie Chen

**Affiliations:** 1 Nanjing Institute of Environmental Sciences, Ministry of Ecology and Environment, Nanjing, 210042 China; 2 State Environmental Protection Key Laboratory on Biodiversity and Biosafety, Nanjing, 210042 China; 3 College of Ecology, Lishui University, Lishui, 323000 China; 4 Lishui Institute for Ecological Economy Research, Lishui, 323000 China

**Keywords:** *Acrossocheilus fasciatus*, LEAP2, Antimicrobial activity, Gene expression, Disulphide bonds

## Abstract

**Background:**

The liver-expressed antimicrobial peptide 2 (LEAP2) plays a pivotal role in the host’s immune response against pathogenic microorganisms. Numerous such antimicrobial peptides have recently been shown to mitigate infection risk in fish, and studying those harboured by the economically important fish *Acrossocheilus fasciatus* is imperative for enhancing its immune responses against pathogenic microorganisms. In this study, we cloned and sequenced LEAP2 cDNA from *A. fasciatus* to examine its expression in immune tissues and investigate the structure–activity relationships of its intramolecular disulphide bonds.

**Results:**

The predicted amino acid sequence of *A. fasciatus* LEAP2 was found to include a signal peptide, pro-domain, and mature peptide. Sequence analysis indicated that *A. fasciatus* LEAP2 is a member of the fish LEAP2A cluster and is closely related to *Cyprinus carpio* LEAP2A. *A. fasciatus* LEAP2 transcripts were expressed in various tissues, with the head kidney exhibiting the highest mRNA levels. Upon exposure to *Aeromonas hydrophila* infection, LEAP2 expression was significantly upregulated in the liver, head kidney, and spleen. A mature peptide of *A. fasciatus* LEAP2, consisting of two disulphide bonds (Af-LEAP2-cys), and a linear form of the LEAP2 mature peptide (Af-LEAP2) were chemically synthesised. The circular dichroism spectroscopy result shows differences between the secondary structures of Af-LEAP2 and Af-LEAP2-cys, with a lower proportion of alpha helix and a higher proportion of random coil in Af-LEAP2. Af-LEAP2 exhibited potent antimicrobial activity against most tested bacteria, including *Acinetobacter guillouiae*, *Pseudomonas aeruginosa*, *Staphylococcus saprophyticus*, and *Staphylococcus warneri*. In contrast, Af-LEAP2-cys demonstrated weak or no antibacterial activity against the tested bacteria. Af-LEAP2 had a disruptive effect on bacterial cell membrane integrity, whereas Af-LEAP2-cys did not exhibit this effect. Additionally, neither Af-LEAP2 nor Af-LEAP2-cys displayed any observable ability to hydrolyse the genomic DNA of *P. aeruginosa*.

**Conclusions:**

Our study provides clear evidence that linear LEAP2 exhibits better antibacterial activity than oxidised LEAP2, thereby confirming, for the first time, this phenomenon in fish.

**Supplementary Information:**

The online version contains supplementary material available at 10.1186/s12917-024-04106-8.

## Background

The liver-expressed antimicrobial peptide 2 (LEAP2) is the second antimicrobial peptide derived from blood [1]. The liver is the primary site of its expression, and it is composed of a cysteine-rich peptide with four conserved cysteine residues [1–3]. LEAP2 antibacterial activity, facilitating the elimination of different pathogenic microorganisms, such as *Bacillus subtilis*, *Bacillus megaterium*, *Micrococcus luteus*, and *Neisseria cinerea*, has been demonstrated [1, 3].

The structure–activity relationship of LEAP2 has been studied in mammals [2, 3]. Henriques et al. [2] demonstrated, through truncation studies, that its C-terminal region is dispensable for membrane binding, whereas its N-terminal (hydrophobic domain) and core regions (cationic domain) are indispensable. Bacterial growth inhibition assays revealed that LEAP2 antibacterial activity is associated with its membrane affinity. Notably, these properties are similar for its oxidised and linear forms, indicating that disulphide bonds are not crucial for bactericidal activity. However, Hocquellet et al. [3] observed that linear LEAP2 has greater antibacterial activity against *B. megaterium* than oxidised LEAP2 and that linear, rather than oxidised, LEAP2 is more effective at penetrating *B. megaterium* membranes. LEAP2 binding to plasmid DNA was also evaluated by Hocquellet et al. using a gel hysteresis assay, wherein the linear form exhibited a DNA-binding effect three times greater than that of the oxidised form [3]. In summary, the impact of structure on LEAP2 antibacterial activity is significant, but the effect of disulphide bonds on antibacterial activity remains inconclusive, owing to inconsistent findings across various studies.

As in mammals, LEAP2 has also been detected in various fishes [4], birds [5], reptiles [6], and even amphibians [7]. Initially, the identification of LEAP2 was limited to *Oncorhynchus mykiss* [8], however, subsequent research efforts have led to its isolation and characterization in a diverse range of fish species [9–18]. In contrast to the singular LEAP2 homologue present in mammals, certain fish species, such as *O. mykiss*, *Larimichthys crocea*, and *Cyprinus carpio*, have multiple homologues, typically categorised as LEAP2A, LEAP2B, and LEAP2C [8, 16, 18]. The expression patterns of LEAP2 exhibit variation across different fish species. For example, *Trachinotus ovatus* LEAP2 is widely distributed, with the highest and lowest expression levels observed in the liver and intestine, respectively [9]. Conversely, in *Larimichthys crocea*, transcript levels in the liver are significantly lower than those in the intestine [16]. The expression of LEAP2 exhibits a robust correlation with the immune response of fish in relation to pathogenic organisms [9–12, 19]. For instance, in *Boleophthalmus pectinirostris*, liver, spleen, kidney, and gill LEAP2 expression is immediately upregulated upon infection with *Edwardsiella tarda* [19].

Fish LEAP2 has antibacterial activity against both gram-positive and gram-negative bacteria [9–12, 19]. For example, purified recombinant *T. ovatus* LEAP2 exerts significant inhibitory effects on *E. tarda* and *Streptococcus agalactiae in vitro* growth. In vivo, the overexpression of *T. ovatus* LEAP2 significantly reduces *E. tarda* and *S. agalactiae* colonisation in fish tissues [9]. Nevertheless, research on LEAP2 structure–activity relationships in fish, specifically regarding the impact of intramolecular disulphide bonds on its antibacterial activity, is lacking.

*Acrossocheilus fasciatus*, of the order Cypriniformes and family Cyprinidae, is a freshwater fish species with notable economic value. It is extensively distributed in streams across southern China; however, its production is impeded by diseases, similar to that with other cultivated fish. Antimicrobial peptides (AMPs) play a crucial role in fish innate immunity, mitigating infection risks, and numerous AMPs have recently been identified [20]. Therefore, studying *A. fasciatus* AMPs is imperative to enhance its immune response against pathogenic microorganisms. Here, we cloned the LEAP2 gene from *A. fasciatus* and examined its expression in immune tissues under normal and infectious conditions. Additionally, we investigated the structure–activity relationships of LEAP2 intramolecular disulphide bonds.

## Results

### Molecular characterisation of A. Fasciatus LEAP2

**Fig. 1:**
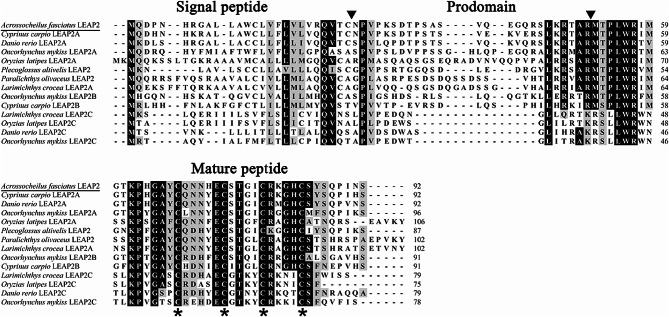
Amino acid sequences of *Acrossocheilus fasciatus* LEAP2 and its homologues based on multiple alignments. A 70% threshold was employed for shading, with grey indicating similar residues, black indicating identical residues, and ‘-’ indicating alignment gaps. The predicted cleavage site for the signal peptide or mature peptide is designated with ‘↓’, and the four conserved cysteine residues in the mature peptide are denoted by ‘*’. Accession numbers of the sequences used are listed in the Fig. 2 legend

The cDNA sequence of *A. fasciatus* LEAP2 was submitted to the GenBank database and assigned the accession number OR046991. The open reading frame of *A. fasciatus* LEAP2, which encompasses 279 nucleotides, was computationally predicted to encode a polypeptide consisting of 92 amino acids. The LEAP2 protein is composed of a signal peptide, a prodomain, and a mature peptide. The anticipated molecular weight of the mature LEAP2 peptide was 4.6 kDa, with a predicted isoelectric point of 8.88. Upon conducting an alignment analysis of *A. fasciatus* LEAP2 with various teleost LEAP2 proteins, it was observed that the functionally mature peptide was conserved among teleosts, while the signal peptide and prodomain displayed variability. All fish LEAP2 proteins also contained four conserved cysteine residues (Fig. 1).

**Fig. 2:**
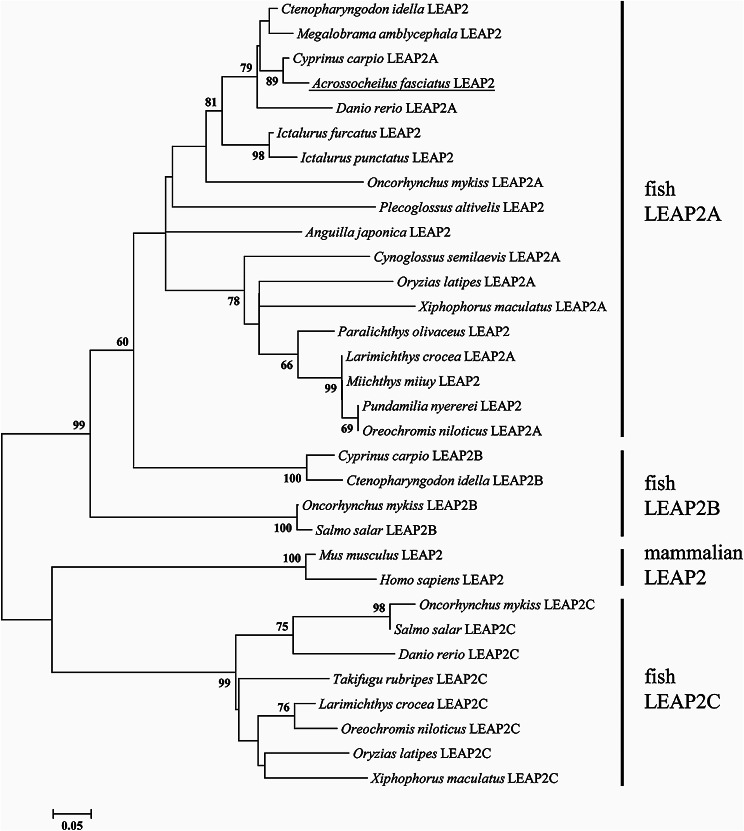
Phylogenetic (Neighbor-joining) analysis of complete amino acid sequences of LEAP2 using the MEGA X program. The values at the forks indicate the percentage of trees in which this grouping occurred after bootstrapping (1000 replicates; shown only when > 60%). The scale bar shows the number of substitutions per base. Accession numbers of sequences used are as follows: *Ctenopharyngodon idella* LEAP2 (FJ390414); *Ctenopharyngodon idella* LEAP2B (KT625603); *Megalobrama amblycephala* LEAP2 (JQ344324); *Cyprinus carpio* LEAP2A (KC551971); *Cyprinus carpio* LEAP2B (KC551972); *Oncorhynchus mykiss* LEAP2A (NM_001124464); *Oncorhynchus mykiss* LEAP2B (NM_001124465); *Oncorhynchus mykiss* LEAP2C (GQ870279); *Pundamilia nyererei* LEAP2 (XM_005750928); *Larimichthys crocea* LEAP2A (KJ024787); *Larimichthys crocea* LEAP2C (KJ024789); *Plecoglossus altivelis* LEAP2 (KJ412462); *Paralichthys olivaceus* LEAP2 (EU586111); *Oryzias latipes* LEAP2A (XM_004079958); *Oryzias latipes* LEAP2C (XM_004074820); *Xiphophorus maculatus* LEAP2A (XM_005806413); *Xiphophorus maculatus* LEAP2C (XM_005810019); *Ictalurus furcatus* LEAP2 (AY845142); *Ictalurus punctatus* LEAP2 (AY845141); *Takifugu rubripes* LEAP2C (XM_011604959); *Danio rerio* LEAP2A (BC162807); *Danio rerio* LEAP2C (XM_009295063); *Salmo salar* LEAP2B (XM_014129528); *Salmo salar* LEAP2C (GQ870280); *Oreochromis niloticus* LEAP2A (XM_003457723); *Oreochromis niloticus* LEAP2C (XM_013270789); *Anguilla japonica* LEAP2 (KP893812); *Cynoglossus semilaevis* LEAP2A (XM_008320540); *Cynoglossus semilaevis* LEAP2A (XM_008324342); *Miichthys miiuy* LEAP2 (KJ000088); *Mus musculus* LEAP2 (BC089593); and *Homo sapiens* LEAP2 (BC070199)

Phylogenetic analysis produced a tree in which teleost LEAP2 sequences were clustered into three distinct groups, LEAP2A, LEAP2B, and LEAP2C, which were found to be separate from the mammalian group (Fig. 2). *A. fasciatus* LEAP2 was identified as a member of the LEAP2A group and was found to be more closely related to LEAP2A of *C. carpio*.

### Constitutive A. fasciatus LEAP2 gene expression

**Fig. 3:**
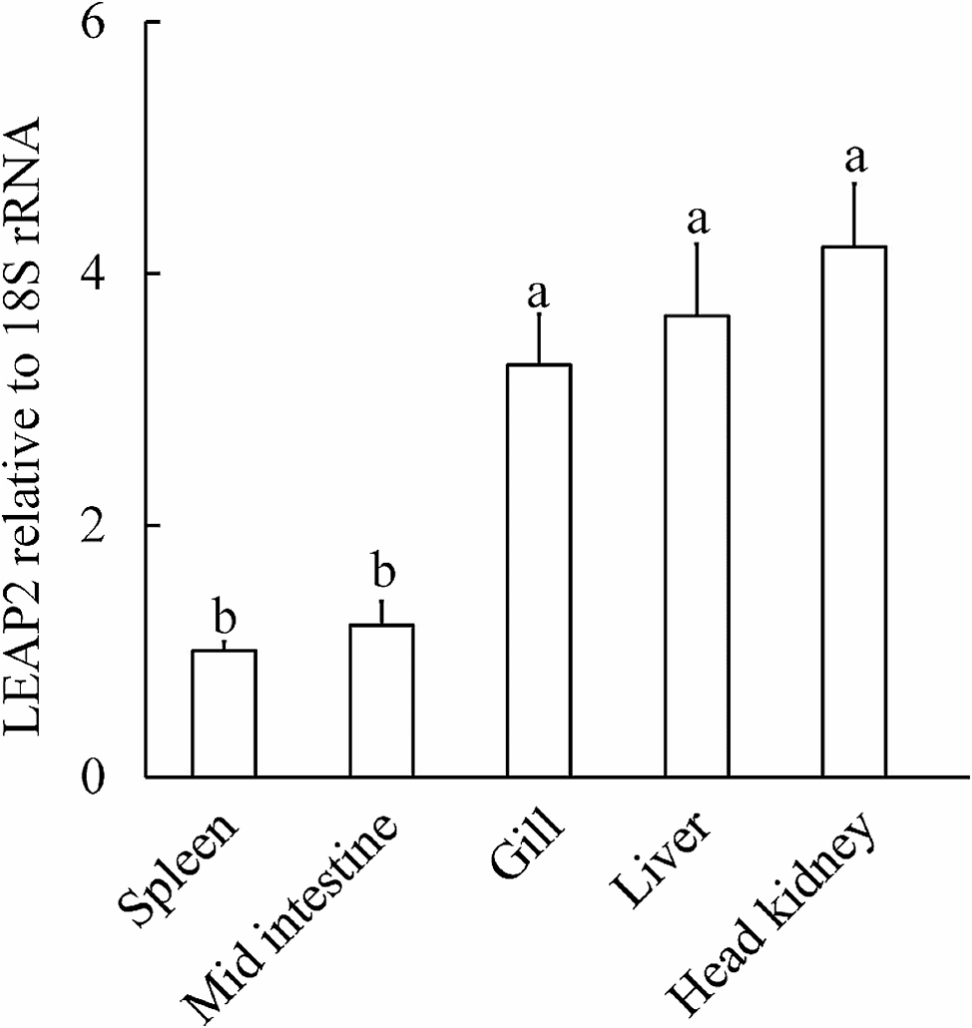
Constitutive expression of LEAP2 in various tissues of *Acrossocheilus fasciatus* based on RT-qPCR. Normalisation of LEAP2 transcript levels was performed with respect to 18 S rRNA. The outcomes are presented as means ± SEMs, with a sample size of *n* = 4

RT-qPCR was used to evaluate constitutive LEAP2 gene expression across various tissues of *A. fasciatus*. The results revealed a discernible expression pattern in multiple tissues, with the head kidney, liver, and gill exhibiting the highest expression, whereas the mid-intestine and spleen displayed relatively lower expression (Fig. 3). Notably, *A. fasciatus* LEAP2 expression in the head kidney was 4.18-fold higher than that in the spleen.

### A. Fasciatus LEAP2 gene expression in response to A. Hydrophila

**Fig. 4:**
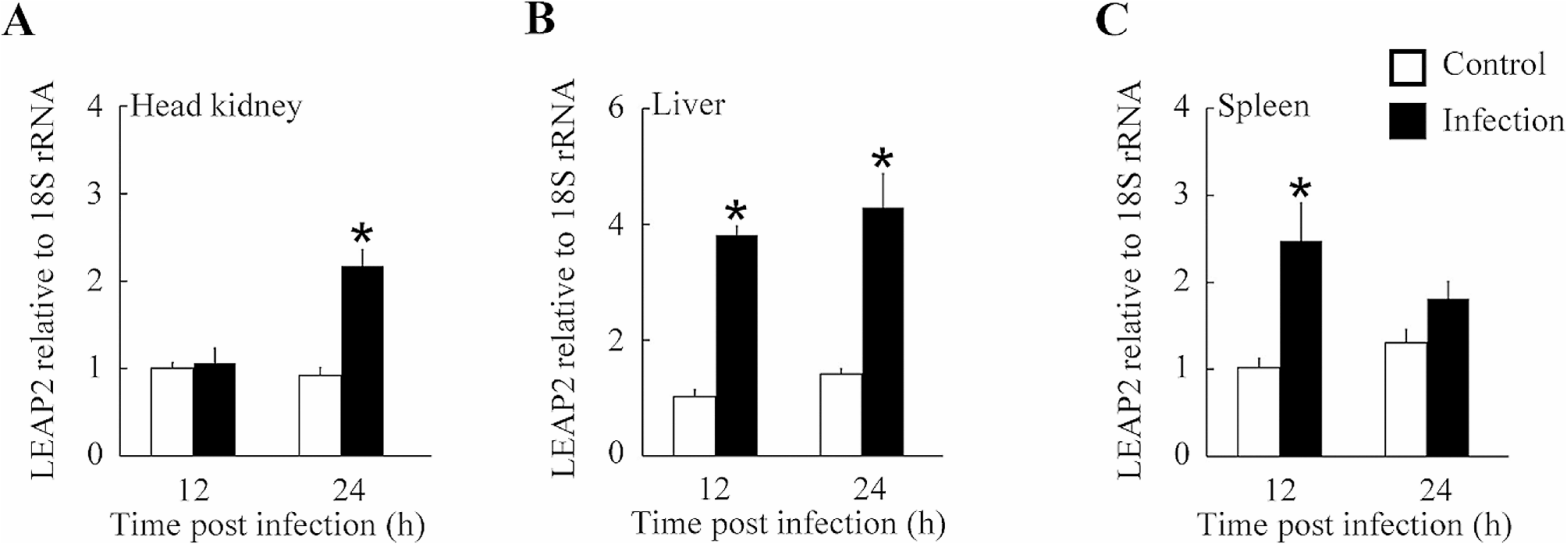
Changes *Acrossocheilus fasciatus* LEAP2 gene expression in immune tissues following *Aeromonas hydrophila* infection. Normalisation of *A. fasciatus* LEAP2 transcript levels was performed with respect to 18 S rRNA. The outcomes are presented as means ± SEMs, with a sample size of *n* = 4. *P < 0.05

In response to *A. hydrophila* challenge, *A. fasciatus* LEAP2 expression was significantly upregulated in all immune tissues examined, as depicted in Fig. 4. Specifically, *A. fasciatus* LEAP2 transcript levels were upregulated at 12 hpi in the liver and spleen (Fig. 4b, c), whereas this occurred at 24 hpi in the head kidney (Fig. 4a). The highest *A. fasciatus* LEAP2 expression levels were detected in the head kidney and liver at 24 hpi and in the spleen at 12 hpi. Regarding peak expression, *A. fasciatus* LEAP2 mRNA levels were upregulated 3.03-fold, 2.43-fold, and 2.36-fold, respectively in the liver, spleen, and head kidney after the challenge.

### The structure of Af-LEAP2-cys and Af-LEAP2

**Fig. 5:**
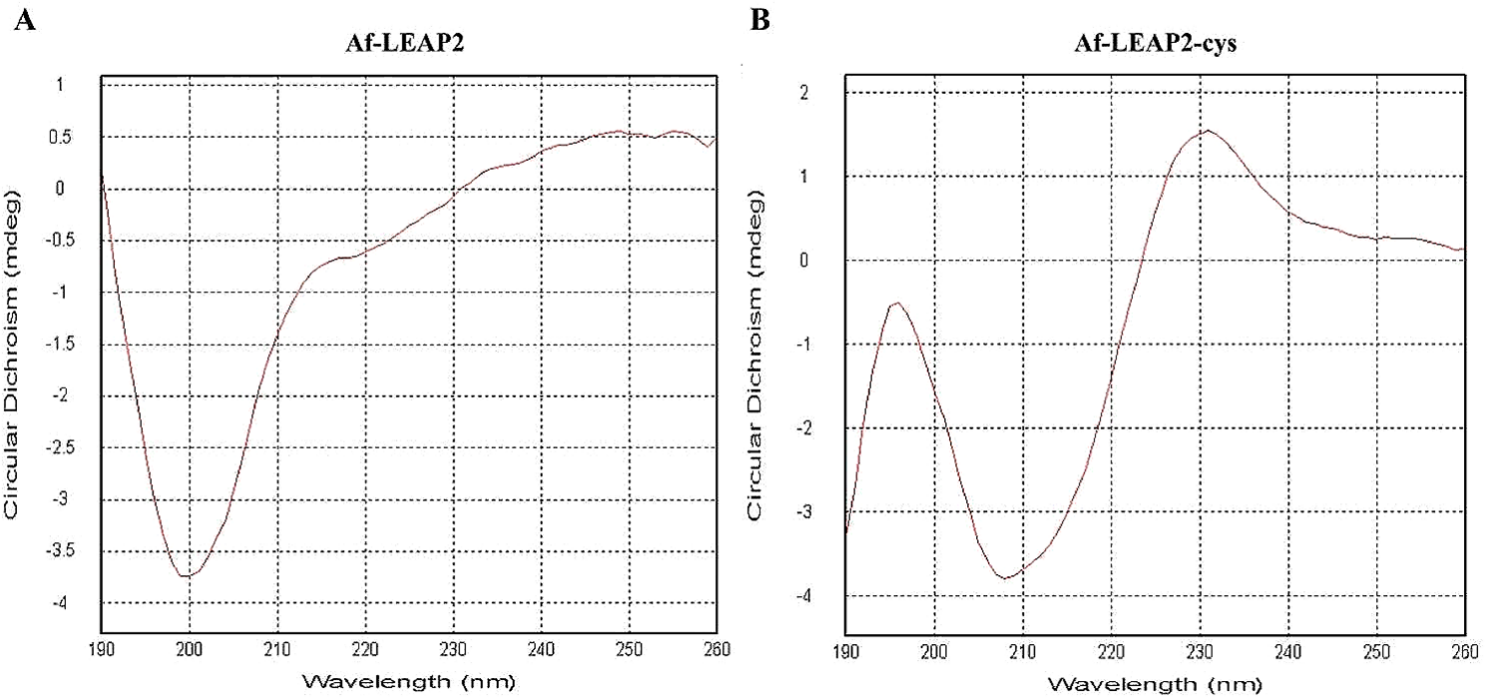
The circular dichroism spectra of of Af-LEAP2 and Af-LEAP2-cys. (**a**) Af-LEAP2, (**b**) Af-LEAP2-cys

A comparative structural study of Af-LEAP2 and Af-LEAP2-cys was performed using circular dichroism spectroscopy. Spectral deconvolution revealed that the secondary structure of Af-LEAP2 was composed of alpha helix (6.6%), antiparallel (38.5%) and parallel (5.2%) sheets, beta turns (22.3%) and random coil (37.6%) (Fig. 5A). In contrast, the secondary structure composition of Af-LEAP2-cys increased the proportion of alpha-helix (8.0%), decreased the proportion of random coil (32.6%), and the proportions of anti-parallel and parallel sheets or beta-turns were 38.9%, 5.2% and 19.8%, respectively (Fig. 5B).

### In vitro antibacterial activity of Af-LEAP2-cys and Af-LEAP2

**Fig. 6:**
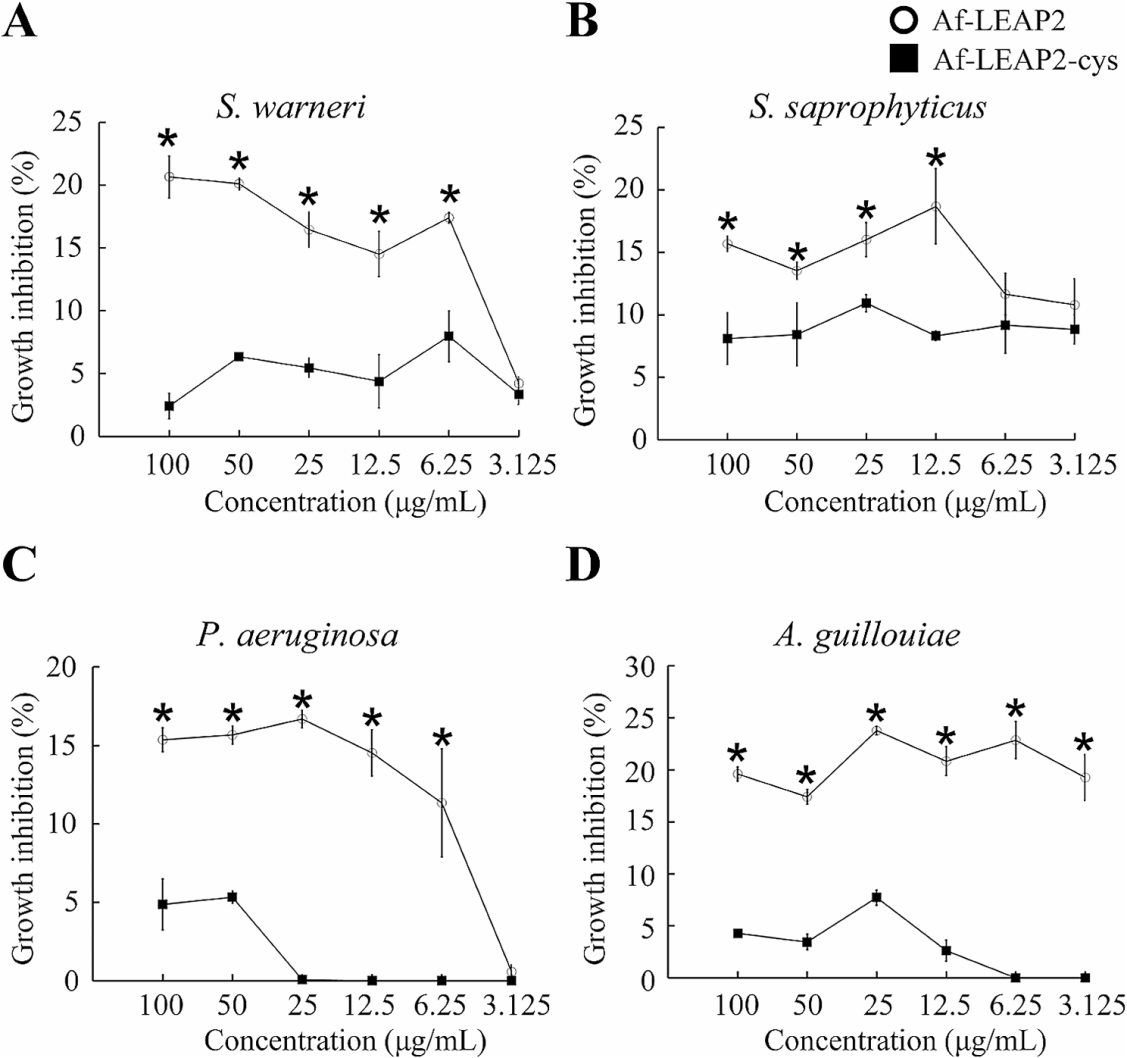
In vitro antibacterial activity of Af-LEAP2 and Af-LEAP2-cys. (**a**) *Staphylococcus warneri*, (**b**) *Staphylococcus saprophyticus*, (**c**) *Pseudomonas aeruginosa*, (**d**) *Acinetobacter guillouiae*. Data are expressed as means ± SEMs of three independent experiments. *P < 0.05

Af-LEAP2 exhibited antibacterial activity against four bacterial strains, *A. guillouiae*, *P. aeruginosa*, *S. saprophyticus*, and *S. warneri*. However, no antibacterial activity was observed against *A. hydrophila* or *E. coli*. In contrast, Af-LEAP2-cys demonstrated either weak or no antibacterial activity against all six bacterial strains at concentrations of 3.125 to 100 µg/mL. As shown in Fig. 6, Af-LEAP2 exhibited a significantly higher inhibitory effect against four bacterial strains, namely *A. guillouiae*, *P. aeruginosa*, *S. saprophyticus*, and *S. warneri*, compared to Af-LEAP2-cys.

### Effects of Af-LEAP2 and Af-LEAP2-cys on P. Aeruginosa cell membrane integrity

**Fig. 7:**
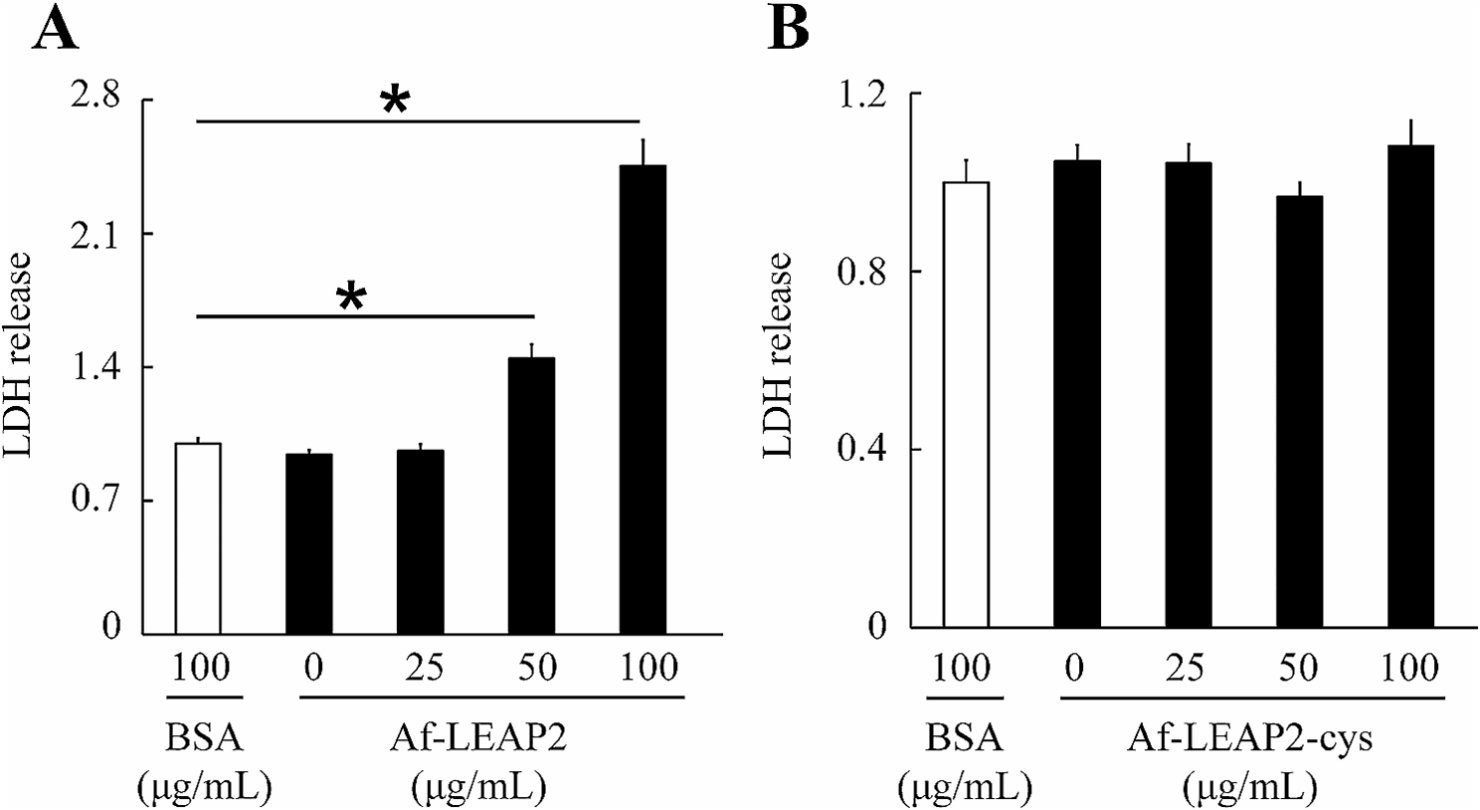
Effects of Af-LEAP2 and Af-LEAP2-cys on the cell membrane integrity in *Pseudomonas aeruginosa*. *P. aeruginosa* was mixed with 0, 25, 50, or 100 µg/mL of Af-LEAP2 and Af-LEAP2-cys for 2 h. Microplate readers were used to measure the absorbance at 490 nm. The negative control comprised bovine serum albumin. Data are expressed as means ± SEMs of three independent experiments. *P < 0.05

To evaluate the effect of Af-LEAP2 and Af-LEAP2-cys on *P. aeruginosa* cell membrane integrity, an LDH release assay was conducted. Results revealed a significant increase in bacterial cell LDH release with 50 and 100 µg/mL of Af-LEAP2 in comparison to that with the BSA control, indicating its disruptive effect on bacterial cell membrane integrity. However, Af-LEAP2-cys did not induce a significant increase in bacterial cell LDH release relative to that with the BSA control, implying that it did not affect bacterial cell membrane integrity (Fig. 7).

### Bacterial gDNA hydrolysis by Af-LEAP2 and Af-LEAP2-cys

**Fig. 8:**
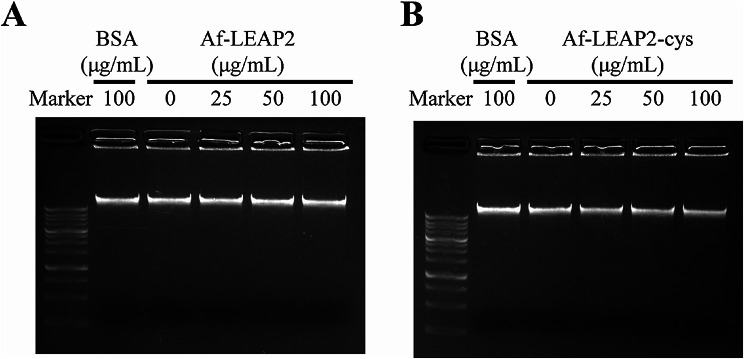
Bacterial genomic DNA hydrolytic activity of Af-LEAP2 and Af-LEAP2-cys. Af-LEAP2 (**a**) or Af-LEAP2-cys (**b**), at 25, 50, and 100 µg/mL, were incubated with of *Pseudomonas aeruginosa* genomic DNA for 30 min, and genomic DNA was analysed via electrophoresis. Bovine serum albumin was used as the negative control. Representative examples of three independent experiments are shown

To assess the effects of Af-LEAP2 and Af-LEAP2-cys on DNA hydrolysis, gDNA extracted from *P. aeruginosa* was treated with Af-LEAP2 or Af-LEAP2-cys, followed by agarose gel electrophoresis. Neither Af-LEAP2 nor Af-LEAP2-cys exhibited any discernible capacity to hydrolyse *P. aeruginosa* gDNA, as evidenced by the unaltered intensity of the gDNA bands (Fig. 8).

## Discussion

LEAP2, a cationic AMP characterised by its high cysteine content and small size, was the second AMP identified in the vertebrate liver, and it has a significant influence on the host innate immune system [7]. Although the structure–activity relationship of LEAP2 has been investigated in mammals [2, 3], the impact of its structure on antibacterial activity is of particular interest. Disulphide bonds and their influence on antibacterial activity remain uncertain due to inconsistent findings across multiple studies. Here, the cDNA sequence of a putative *A. fasciatus* LEAP2 gene was determined. The encoded LEAP2 consists of a signal peptide, prodomain, and mature peptide. Similar to that in most teleosts, the mature LEAP2 peptide in *A. fasciatus* contains four conserved cysteine residues forming two pairs of disulphide bonds. Our phylogenetic analysis suggests that *A. fasciatus* LEAP2 is a member of the fish LEAP2A cluster with the closest phylogenetic relationship to *C. carpio* LEAP2A. The collective phylogenetic and amino acid sequence results suggest that this molecule might have played a significant role in the evolutionary history of vertebrates.

Our study revealed that *A. fasciatus* LEAP2 is expressed constitutively in various tissues of healthy fish, particularly in the head kidney, liver, and gills. These findings are consistent with those of previous investigations on different fish species [9, 17, 21]. Notably, LEAP2 expression in *T. ovatus* is high in the liver, spleen, head kidney, and gills [9], whereas in *O. mykiss*, LEAP2A, and LEAP2B are constitutively expressed only in the liver [8]. Our investigation revealed that following *A. hydrophila* infection, *A. fasciatus* LEAP2 expression is upregulated in all examined tissues, consistent with previous studies on other fish species [8–12, 14, 16, 18, 19, 22, 23]. For example, in *B. pectinirostris*, the liver, spleen, kidney, and gills exhibit an immediate increase in LEAP2 expression upon *E. tarda* infection [19]. Similarly, in *Plecoglossus altivelis*, LEAP2 expression is increased in the liver, gills, spleen, kidney, and heart in response to *Vibrio anguillarum* infection [22]. These findings suggest that LEAP2 plays a crucial role in the innate immune response.

In our study, spectral deconvolution shows differences between the secondary structures of Af-LEAP2 and Af-LEAP2-cys, with a lower proportion of alpha helix (6.6%) and a higher proportion of random coil (37.6%) in Af-LEAP2. In contrast, Af-LEAP2-cys had a higher proportion of alpha-helix (8.0%) and a lower proportion of random coil (32.6%), suggesting that Af-LEAP2 is more structurally disordered than Af-LEAP2-cys. Hocquellet et al. [3] performed a comparative structural analysis of the linear and recombinant forms of human LEAP-2 using one-dimensional 1 H NMR spectroscopy. The spectra showed clear differences in the presence or absence of disulfide bridges. In the linear form, the narrow spectral range of the amide region (7.8–8.6 ppm) and the absence of the Ha resonance above the water signal (4.8 ppm) indicate that the peptide is disordered in the absence of disulfide bridges. Conversely, the recombinant peptide adopts a well-defined conformation due to the formation of two disulfide bridges. All of these results suggest that linear LEAP2 is more structurally disordered.

Recently, in vitro investigations have ascribed direct antibacterial activity to fish LEAP2 [14, 16, 17, 19, 22]. For example, synthetic peptides derived from *B. pectinirostris* LEAP2 were found to have distinct antimicrobial activity against *Vibrio vulnificus* and *Vibrio alginolyticus in vitro* [19]. In this study, Af-LEAP2 exhibited potent antimicrobial activity against most tested bacteria, including *A. guillouiae*, *P. aeruginosa*, *S. saprophyticus*, and *S. warneri*. In contrast, Af-LEAP2-cys demonstrated weak or no antibacterial activity against all tested bacteria. Similarly, Hocquellet et al. [3] observed that linear LEAP2 has stronger antibacterial activity than oxidised LEAP2 in mammals. However, Henriques et al. [2] showed that oxidised LEAP2 has an antibacterial activity similar to that of linear LEAP2.

For a more comprehensive study on the potential variability in the antibacterial activity of oxidised LEAP2 versus linear LEAP2, we tested both for their effects on cell membrane structural integrity and their ability to catalyse bacterial gDNA hydrolysis. This provided evidence that Af-LEAP2, but not Af-LEAP2-cys, disrupts bacterial cell membrane integrity. Additionally, neither Af-LEAP2 nor Af-LEAP2-cys displayed any observable ability to hydrolyse *P. aeruginosa* gDNA. AMPs typically exert their effects either via membrane permeabilisation through pore formation or translocation across the membrane to gain access to the interior of the cell and attack internal targets [24, 25]. Our findings indicate that fish LEAP2 antibacterial activity is primarily attributable to its ability to insert itself into bacterial cell membranes and disrupt their structural integrity. Regarding the higher capacity of linear LEAP2 to disrupt the bacterial cell membrane compared to that of oxidised LEAP2, it was hypothesised that these peptides differ in their amphiphilicity. Specifically, linear LEAP2 has a more flexible and less globular structure than oxidised LEAP2, which is constrained by disulphide bonds. This structural difference might have facilitated the insertion of linear LEAP2 into the hydrophobic core of the membrane. Henriques et al. [2] observed that linear LEAP2 results in more pronounced membrane insertion than oxidised LEAP2, as evidenced by alterations in the membrane dipolar potential and Trp fluorescence properties. The globular structure of oxidised LEAP2 is more conducive to receptor binding, and in a number of recent studies, LEAP2 can bind to the MOSPD2 receptor and regulate immune function [26]; in addition, LEAP2 can also bind to the GHSR1a and regulate growth and development [27].

The escalating frequency of bacterial resistance towards currently available antibiotics poses a significant challenge, underscoring the urgency for innovative anti-infective strategies. AMPs emerge as promising candidates to complement conventional antibiotics. Boasting flexibility, AMPs possess an extensive sequence diversity and can be tailored to exhibit either broad-spectrum or targeted antimicrobial activity. Nevertheless, several hurdles, including toxicity, stability, and bacterial resistance, must be addressed before AMPs can be developed for widespread application. Rational AMP design offers a promising approach to addressing these challenges and may pave the way for their utilization as novel antimicrobials. Fázio et al. [28] demonstrated the importance of the β-turn structure in influencing the biological activities of gomesin. Through the design of analogs that modified the disulfide bonds of the peptide, the authors observed that enhancing the β-like conformation of the peptide enhanced its antimicrobial activity, whereas analogs with unrestrained structures exhibited diminished activity. Du et al. [29] developed AcrAP1 and AcrAP2 analogs based on peptides from scorpion *Androctonus crassicauda* venom, which exhibited enhanced cationicity. These derivatives demonstrated heightened antimicrobial efficacy and a wider range of activity, while also displaying the ability to influence the proliferation of various human cancer cell lines, a characteristic not observed in the original peptides. In our research, the linear LEAP2 exhibits a more flexible and less spherical structure compared to its oxidized form. This structural difference enhances the antibacterial activity of linear LEAP2, making it more effective against bacterial infections.

## Conclusion

In summary, a LEAP2 homologue from *A. fasciatus* was characterised, and its expression was found to be higher in the head kidney and liver of *A. fasciatus*. Linear LEAP2 (Af-LEAP2) had stronger antibacterial activity than oxidised LEAP2 (Af-LEAP2-cys), and Af-LEAP2, but not Af-LEAP2-cys, disrupted bacterial cell membrane integrity. Our study provides clear evidence that linear LEAP2 has better antibacterial activity than oxidised LEAP2, thereby confirming, for the first time, this phenomenon in fish.

## Methods

### Experimental fish

The collection of all samples was conducted with due permission in compliance with the local license. The methods employed were executed in adherence to the pertinent guidelines and regulations stipulated in the ethics approval and consent to participate section, as well as with the endorsement of the Ethics Committee of Lishui University (Permit No. AREC-LSU202212–006) and ARRIVE guidelines.

*A. fasciatus* (20 ± 3.0 g) was obtained from Longquan Fuxi Agricultural Development Co, LTD, Lishui City, China. Fish were reared under controlled conditions, in 100 L tanks equipped with a recirculation system and a temperature-controlled environment of 20–22 °C. The fish were fed a commercial diet twice daily, with a 2-week acclimation period prior to commencing experiments.

### Molecular characterisation of A. Fasciatus LEAP2 cDNA

The cDNA sequence of *A. fasciatus* LEAP2 was obtained from transcriptome data of the *A. fasciatus* liver. Sequence similarity to other known sequences was evaluated using BLAST (http://blast.ncbi.nlm.nih.gov/Blast.cgi). Signal peptide cleavage sites were predicted using SignalP (http://www.cbs.dtu.dk/services/SignalP). The mature peptide sequence of *A. fasciatus* LEAP2 was determined using ProP 1.0 (https://services.healthtech.dtu.dk/services/ProP-1.0/). ClustalW (http://clustalw.ddbj.nig.ac.jp/) was used to analyse multiple alignments, whereas MEGA version X was used for phylogenetic and molecular evolutionary analyses.

### Basal and inducible A. fasciatus LEAP2 gene expression in various tissues

To conduct an analysis of tissue differential expression, a total of four healthy individuals of *A. fasciatus* were utilized to obtain spleen, gill, liver, head kidney, and mid-intestine tissues. In order to perform bacterial challenge experiments, 16 *A. fasciatus* individuals were evenly distributed into two groups, namely the infection (*n* = 8) and control (*n* = 8) groups. The bacterial challenge experiments were conducted in accordance with established protocols [30]. Specifically, *A. hydrophila* was collected during the mid-logarithmic phase, washed thrice in sterile PBS, and standardized to a final inoculum of 1.0 × 10^5^ colony-forming units (CFU)/mL. The infection group of *A. fasciatus* was subjected to intraperitoneal injection with 1.0 × 10^4^ CFU of live *A. hydrophila* per fish, while the control group was administered PBS. At 12 and 24 h after infection (hpi), four fish were selected from each group, and the liver, spleen, and head kidney were collected and conserved at − 80 °C until subsequent examination. All fish were anaesthetised with 0.1 g/L tricaine methanesulphonate (MS-222) prior to dissection.

### RT-qPCR

Total RNA was extracted from the tissues using Beyozol (Beyotime, Shanghai, China). Subsequently, BeyoRT™ III First Strand cDNA Synthesis Kit with gDNA EZeraser (Beyotime) was employed to synthesize first-strand cDNA. Gene-specific primers were designed based on LEAP2 and 18 S rRNA sequences (Table 1). The qPCR assay was performed with BeyoFast™ SYBR Green qPCR Mix (2X) (Beyotime) using the CFX96 real-time PCR detection system (Bio-Rad, Hercules, USA). The amplification procedure were as follows: 5 min at 95 °C, 40 cycles of 30 s at 95 °C, 30 s at 60 °C, and 30 s at 72 °C, followed by melting curve analyses. Threshold cycle (Ct) for LEAP2 were normalized to 18 S rRNA (GenBank accession number: OM919510) using the 2^−ΔΔCt^ method [31].

**Table 1:** Sequences of oligonucleotide primers

Gene	Primers	Sequence (5′-3′)	Amplicon size (bp)
LEAP2	LEAP2-t(+)	TGCAGGAAGGTCAAAGGTCA	154
	LEAP2-t(-)	ATCGGTTGGCTGTAGGAACA	
18 S rRNA	18 S rRNA-t(+)	CCGAGATCCAACTACGAGCT	247
	18 S rRNA-t(-)	AGAAACGGCTACCACATCCA	

### Peptide synthesis and circular dichroism spectroscopy analyses

The *A. fasciatus* LEAP2 mature peptide (MTPLWRIMGTKPHGAYCQNNHECSTGICRKGHCSYSQPINS), comprising two disulphide bonds (Af-LEAP2-cys), was chemically synthesised with purity exceeding 95% using SynPeptide in Shanghai, China. Furthermore, the linear *A. fasciatus* LEAP2 mature peptide (Af-LEAP2) form was synthesised chemically with a purity exceeding 95% using SynPeptide. Circular dichroism spectra were recorded using a Chirascan with a 1 mm pathlength cell at 0.2 mg/mL peptide concentration in 10 mM phosphate sodium, 30 mM NaCl, *p*H 6. Ten spectra were recorded and accumulated from 190 to 260 nm every 1 nm with a 1 nm bandwidth at room temperature. The final spectra of Af-LEAP2 and Af-LEAP2-cys were deconvoluted using the CDNN program.

### Antibacterial assay

The antibacterial activities of both were evaluated against a variety of bacteria, including *A. hydrophila*, *Acinetobacter guillouiae*, *Pseudomonas aeruginosa*, *Staphylococcus saprophyticus*, *Staphylococcus warneri*, and *Escherichia coli*, using a modified two-fold microdilution method, as previously described [7]. The percentage inhibition of bacterial growth was assessed by quantifying the absorbance of bacterial sedimentation at 600 nm using a microplate reader (Varioskan Flash Multimode Reader; Thermo Fisher Scientific, USA).

### Lactate dehydrogenase (LDH) release assay

To evaluate the impact of the peptides on bacterial cell membrane integrity, we used an LDH Release Assay Kit (Beyotime), as per the manufacturer’s instructions, with minor modifications. *P. aeruginosa* cells, at 1 × 10^9^ CFUs, were pelleted and resuspended in 200 µL of PBS. The bacteria were then treated with Af-LEAP2-cys or Af-LEAP2 at 25, 50, and 100 µg/mL and incubated at 37 °C for 2 h. The samples were then centrifuged at 8000 × *g* for 2 min, and 120 µL of the supernatant was transferred to a 96-well plate. Each well was supplemented with 60 µL of the LDH detection working solution, followed by thorough mixing and incubation at room temperature for 30 min. The absorbance was then quantified at 490 nm.

### DNA degradation assay

The hydrolysis of *P. aeruginosa* genomic DNA (gDNA) mediated by Af-LEAP2-cys and Af-LEAP2 was evaluated using established methodologies [7]. *P. aeruginosa* gDNA was extracted using an Universal Genomic DNA Purification Mini Spin Kit (Beyotime) and quantified using a NanoDrop 2000 instrument (Thermo Fisher Scientific, Wilmington, DE, USA). Subsequently, gDNA was subjected to incubation with varying concentrations of Af-LEAP2-cys or Af-LEAP2, specifically 25, 50, and 100 µg/mL. The mixture was incubated for a duration of 30 min at room temperature, after which 1.0% agarose gels were utilized for analysis.

### Statistical analysis

Data are presented as the mean (SEM), and statistical analyses were performed using SPSS (version 13.0 (SPSS Inc., Chicago, USA). Expression data were analysed using one-way ANOVA and the least significant difference post-hoc test, with statistical significance defined as *P*-values less than 0.05.

### Electronic supplementary material

Below is the link to the electronic supplementary material.

Supplementary Material 1

## Data Availability

The *A. fasciatus* LEAP2 cDNA sequence was submitted to GenBank under accession number OR046991. The datasets used and/or analysed during the current study are available from the corresponding author on reasonable request.

## Declarations

### Novelty statement

We successfully cloned and sequenced the LEAP2 cDNA from *A. fasciatus*. Furthermore, we observed that linear LEAP2 exhibits stronger antibacterial activity in comparison to its oxidised form. This finding marks the first instance of confirmation of this phenomenon among fish species.
