# Supplementary material for: Structure–activity relationships of the intramolecular disulphide bonds in LEAP2, an antimicrobial peptide from Acrossocheilus fasciatus
Source: BMC Vet Res. 2024 Jun 4;20:243. doi: 10.1186/s12917-024-04106-8 (PMC11149183; doi:10.1186/s12917-024-04106-8)
Supplement: Supplementary file 1 — Supplementary Material 1 [file 12917_2024_4106_MOESM1_ESM.docx]

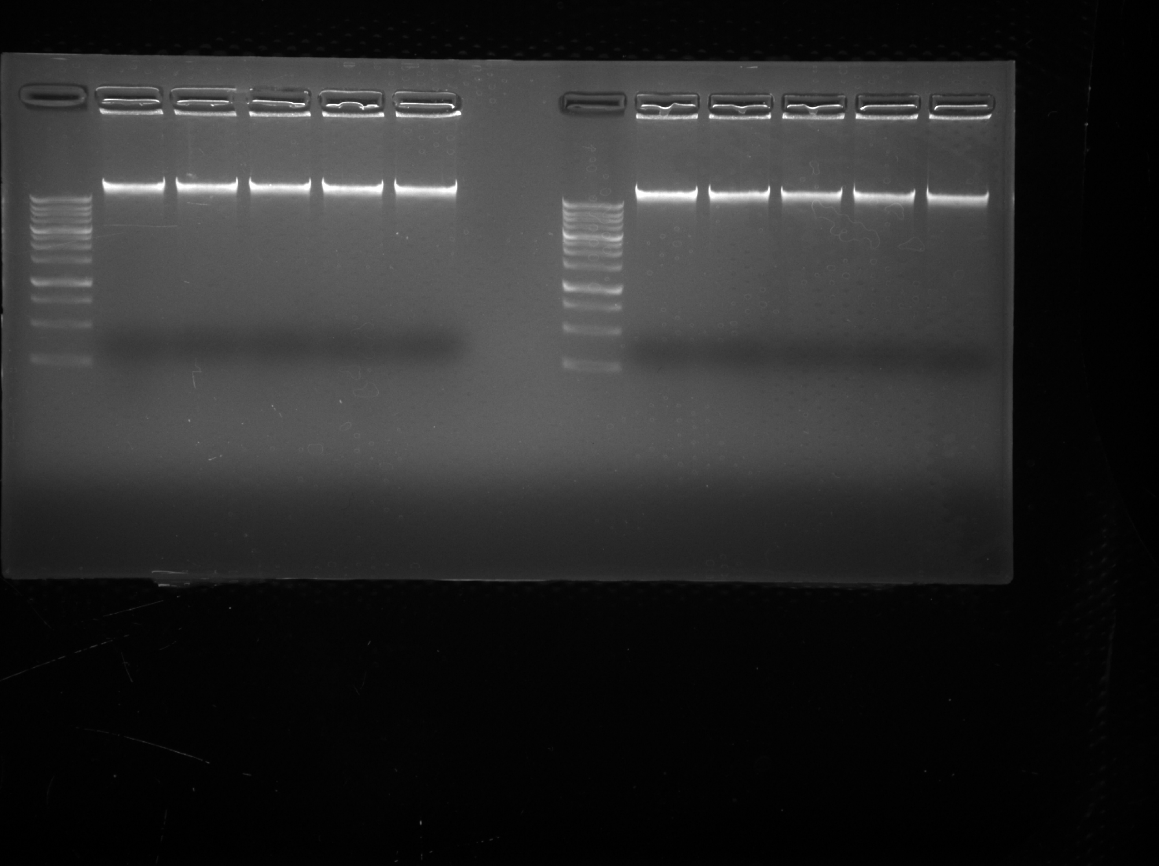


**Figure 8** Bacterial genomic DNA hydrolytic activity of Af-LEAP2 and Af-LEAP2-cys. Af-LEAP2 (left) or Af-LEAP2-cys (right), at 25, 50, and 100 μg/mL, were incubated with of *Pseudomonas aeruginosa* genomic DNA for 30 min, and genomic DNA was analysed via electrophoresis. Bovine serum albumin was used as the negative control. Representative examples of three independent experiments are shown.
